# The Use of Trisodium Citrate to Improve the Textural Properties of Acid-Induced, Transglutaminase-Treated Micellar Casein Gels

**DOI:** 10.3390/molecules23071632

**Published:** 2018-07-04

**Authors:** Hongliang Li, Chang Yang, Chong Chen, Fazheng Ren, Yuan Li, Zhishen Mu, Pengjie Wang

**Affiliations:** 1 Beijing Advanced Innovation Center for Food Nutrition and Human Health, College of Food Science & Nutritional Engineering, China Agricultural University, Beijing 100083, China; l12wpj@cau.edu.cn (H.L.); renfazheng@cau.edu.cn (F.R.); 16828777@163.com (Y.L.); 2 Inner Mongolia Mengniu Dairy (Group) CO., Ltd., Hohhot 750306, China; fdl409@cau.edu.cn (C.Y.); muzhishencau@126.com (Z.M.); 3 Key Laboratory of Functional Dairy, Co-Constructed by Ministry of Education and Beijing Government, Beijing 100083, China; chenchong409@cau.edu.cn; 4 Beijing Laboratory of Food Quality and Safety, Beijing Higher Institution Engineering Research Center of Animal Product, Beijing 100083, China

**Keywords:** trisodium citrate, microbial transglutaminase, casein gels, textural properties, microstructures

## Abstract

In this study, the effect of trisodium citrate on the textural properties and microstructure of acid-induced, transglutaminase-treated micellar casein gels was investigated. Various concentrations of trisodium citrate (0 mmol/L, 10 mmol/L, 20 mmol/L, and 30 mmol/L) were added to micellar casein dispersions. After being treated with microbial transglutaminase (mTGase), all dispersions were acidified with 1.3% (*w*/*v*) gluconodelta-lactone (GDL) to pH 4.4–4.6. As the concentration of trisodium citrate increased from 0 mmol/L to 30 mmol/L, the firmness and water-holding capacity increased significantly. The final storage modulus (G′) of casein gels was positively related to the concentration of trisodium citrate prior to mTGase treatment of micellar casein dispersions. Cryo-scanning electron microscopy images indicated that more interconnected networks and smaller pores were present in the gels with higher concentrations of trisodium citrate. Overall, when micellar casein dispersions are treated with trisodium citrate prior to mTGase crosslinking, the resulted acid-induced gels are firmer and the syneresis is reduced.

## 1. Introduction

Yogurt is a very popular fermented milk-based product worldwide due to its nutritional and sensory properties [1]. However, the acid-induced milk gels often have textural defects such as low firmness (fragile structure) and syneresis (low water holding capacity) during storage or following mechanical damage [2], which greatly reduce their sensory properties. Therefore, the development of acid-induced milk gels with greater firmness and water holding capacity is an important issue.

The firmness and water-holding capacity of acid-induced milk gels are closely related to their network structures. The primary building blocks of acid-induced milk gel network structures are proteins. Microbial transglutaminase (mTGase) is an enzyme widely used to form covalent links between individual protein molecules [3,4,5,6]. The textural properties of acid-induced gels prepared from mTGase-treated milk proteins can be significantly enhanced [2,7]. Unfortunately, even though plenty of work has been carried out regarding the effect of mTGase on the textural properties of acid-induced milk gels, the gel defects including low firmness (fragile structure) and syneresis have not been completely prevented.

The related mechanism of the mTGase-induced changes in the texture of acidified milk protein gels is summarized below [8,9]. The primary building blocks of acid-induced milk gel network structures are caseins [10,11,12], which are cross-linked with calcium phosphate to form association colloids known as casein micelles [12,13,14]. Schorsch and others [15] investigated the effect of the mTGase treatment on the acid gelation of micellar caseins. The mTGase generated covalent bonds between the individual casein molecules on the micellar surface or within the micelles. This inhibited the division of casein micelles even though the solubilization of calcium phosphate upon acidification occurred [16]. In this case, the casein particles could not be adequately rearranged to form more interconnected gel networks.

The addition of calcium-chelating agents (for example, trisodium citrate, sodium phosphate) to micellar casein dispersions has been reported to dissociate the native casein micelles into smaller casein aggregates [12,17,18]. This may change the rearrangement of casein particles during gel formation and the contact between mTGase and caseins, which would alter the crosslink bonds between casein molecules before or during acidification. Therefore, the mTGase-treated smaller casein particles might create different gel network structures compared with the gels from acidified mTGase-treated native casein micelles.

In this study, the effect of the calcium-chelating agent, which is trisodium citrate (TSC), on the textural properties and microstructure of acid-induced, transglutaminase-treated micellar casein gels was investigated with special reference to the related mechanisms of action.

## 2. Results and Discussion

The building blocks of the acid-induced milk gel network structures are proteins, which include approximately 80% of caseins and 20% of whey proteins [10]. Whey proteins were susceptible to mTGase only after being denatured by heat treatment [19]. In addition, the denatured whey proteins play an important role in the textural properties of yogurt, but some denatured whey proteins form particles [20,21,22]. The particle diameters range from about 80 nm to 500 nm [23,24], which was in the particle size range of casein micelles. Therefore, in the complex system consisting of both casein micelles and denatured whey proteins, it would be difficult to distinguish the changes of casein micelles with different treatments. Due to the primary role of casein micelles in the formation of acid-induced milk gels, the whey proteins were not included in the present study, as reported in previous papers [6,15,25,26].

The preparation of micellar casein gels can be divided into three routes which are described in the Materials and Methods section.

In route 1, various amounts of trisodium citrate were added to the casein micelle dispersions before mTGase treatment, which was followed by acidified with GDL. For convenience, the dispersions were first treated with various amounts of trisodium citrate and then treated with mTGase, which are defined as TG, 10-TG, 20-TG, and 30-TG, respectively, where the numbers refer to 0 mmol/L trisodium citrate, 10 mmol/L trisodium citrate, 20 mmol/L trisodium citrate, and 30 mmol/L trisodium citrate, respectively.

In route 2, various amounts of trisodium citrate were added to the casein micelle dispersions after mTGase treatment, which was followed by acidification with GDL. For convenience, the dispersions are first treated with mTGase and then treated with various amounts of trisodium citrate, which are defined as TG, TG-10, TG-20, and TG-30, respectively, where the numbers refer to 10 mmol/L trisodium citrate, 20 mmol/L trisodium citrate, and 30 mmol/L trisodium citrate, respectively. TG refers to samples with no trisodium citrate added.

In route 3, micellar casein dispersions treated with 0 mmol/L trisodium citrate, 10 mmol/L trisodium citrate, 20 mmol/L trisodium citrate, and 30 mmol/L trisodium citrate (in the absence of mTGase) were directly acidified with GDL.

### 2.1. Particle Size of Casein Micelles and Soluble Calcium Contents

Table 1 shows the effects of trisodium citrate and routes on the average diameter of casein particles. Two-way ANOVA revealed that there was a significant effect of routes on the particle sizes. In route 1 or route 3, with increasing trisodium citrate concentrations, the average diameter of particles exhibited an obvious decreasing trend. This suggested that the particle size in casein micelle dispersions became smaller with increasing trisodium citrate concentrations. The soluble calcium concentrations in casein micelle dispersions containing 0 mM trisodium citrate, 10 mM trisodium citrate, 20 mM trisodium citrate, and 30 mM trisodium citrate were 8.6 ± 0.5 mM, 13.3 ± 0.8 mM, 23.7 ± 0.6 mM, and 23.6 ± 0.6 mM, respectively. Similar results can be also found in Reference [27]. It is well-established that calcium-chelating agents can disrupt the micellar framework by removing calcium from the micelles, which leads to the dissociation of casein micelles [17]. Therefore, it was expected that the presence of citrate ions would reduce the particle size of native casein micelles. However, in route 2, the diameter of particles in TG, TG-5, TG-10, TG-20, and TG-30 showed little variation. This may be due to cross-linking of caseins within the particles by mTGase-generated covalent casein networks. Although the colloidal calcium phosphate dissociated from the casein micelles in the presence of citrate ions, this did not disrupt the stability of the mTGase-treated micelles [4,28].

### 2.2. Gelation Kinetics

**Figure 1:**
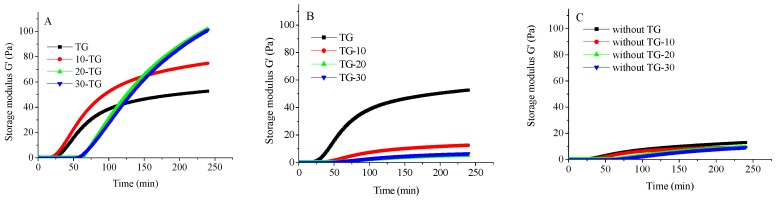
The evolution of the storage modulus as a function of time ((**A**) Route 1; (**B**) Route 2; (**C**) Route 3 (without mTGase)).

The effect of trisodium citrate on the storage modulus (G′) as a function of time after the addition of GDL is shown in Figure 1. As expected, the G′ increased after gelation, followed by a plateau during the gel stage. Similar results have been reported elsewhere [29,30]. It can be seen that trisodium citrate had a significant influence on the acid-induced gelation kinetics of mTGase-treated micellar caseins. In the three routes, gelation time (when G′ ≥ 1) was positively related to the trisodium citrate concentration. This may be because trisodium citrate could dissolve the colloid calcium phosphate in the micelles, which leads to the liberation of phosphate into the soluble phase. Due to the buffering capacity of phosphate, with the increasing concentrations of trisodium citrate, a slower acidification rate of the dispersions occurred [27]. It can also be observed that, in the same trisodium citrate concentration, the final G′ values of gels prepared in route 1 were significantly higher than route 3. This indicated the mTGase-crosslinking played a crucial role in the gel structure. In route 1, the final G′ of the gels was positively related to the trisodium citrate concentrations. As mentioned above, when the amount of trisodium citrate was increased, the native casein micelles dissociated into smaller casein particles. This increased the flexibility of the casein particles, which favored the rearrangement of caseins during gel formation. In addition, the surface area of the smaller casein particles was larger than the native casein micelles, which favored contact between mTGase and caseins. This enhanced the crosslink bonds between casein molecules before or during acidification [31]. Due to these two reasons, in route 1, the final G′ of gels increased with trisodium citrate concentration. However, the final G′ of gels formed in route 2 decreased with increasing concentrations of trisodium citrate. The size of casein particles in route 2 showed little variation in different trisodium citrate concentrations. During acidification, calcium binding or the other non-covalent interactions may have favored the coagulation of individual micelles. Therefore, the presence of the calcium-chelating agent trisodium citrate in route 2 decreased the calcium binding or other non-covalent interactions (especially electrostatic repulsion) between casein particles. This could inhibit the interconnectivity between micelles, which would lead to lower final G′ values of the gels.

### 2.3. The Firmness of Acid-Induced Casein Gels

The results displayed in Table 2 illustrate the effect of trisodium citrate on the firmness of acid-induced micellar casein gels. Two-way ANOVA revealed that there was a significant route effect on the firmness of gels. In the samples without mTGase (route 3), it can be seen that the addition of trisodium citrate had little influence on the firmness of acid-induced casein gels. It was observed that the firmness of gels prepared in route 1 decreased in the following order: 30-TG or 20-TG > 10-TG > TG. These findings suggested that the firmness of GDL-induced micellar casein gels was positively related to the amount of trisodium citrate in route 1. The reasons for this was previously explained for the storage modulus (G′) of gels. It can also be observed that in the same trisodium citrate concentration, the firmness of gels prepared in route 1 was significantly higher than route 3. This indicated that the mTGase-crosslinking played a crucial role in the gel firmness.

The firmness of gels prepared in route 2 decreased with increasing trisodium citrate concentration. These trends are in agreement with the rheological results. It was also found that the firmness of gels prepared from 30-TG was significantly greater than the firmness of gels prepared from TG-30, which was the same as that in the other groups (20-TG versus TG-20 and 10-TG versus TG-10). This indicated that trisodium citrate treatment before the enzyme cross-linking of casein micelles resulted in firmer gels than those treated after cross-linking. However, trisodium citrate treatment after the enzyme cross-linking of casein micelles had no significant influence on gel firmness. The mTGase-treated casein micelles could not be dissociated into smaller particles even though colloidal calcium phosphate can be removed by trisodium citrate [32]. Therefore, the flexibility of the casein particles was not enhanced and the crosslink bonds between casein molecules were fewer in the gels prepared in route 2 than in route 1.

### 2.4. Water Holding Capacity of Acid-Induced Casein Gels

The effect of trisodium citrate on the water-holding capacity of acid-induced micellar casein gels is shown in Table 3. Two-way ANOVA revealed that there was a significant effect of routes on the water-holding capacity of gels. It can be seen that, in the presence of trisodium citrate, little water in the gels prepared in route 1 was expelled by centrifugation. However, more than 30% of water was expelled in the gels prepared in route 2. This indicated that the gels prepared from the smaller particles in route 1 exhibited greater water retention than the gels prepared in route 2. This was consistent with the results of gel firmness since weak gels tend to flow and are more prone to shrinkage and subsequent expulsion of whey [33]. It can be also observed that, in the same trisodium citrate concentration, the water holding capacity of gels prepared in route 1 was significantly higher than in route 3. This indicated that the mTGase-crosslinking played a crucial role in improving the water-holding capacity.

### 2.5. Microstructure and Degree of Cross-Linking of Acid-Induced Casein Gels

**Figure 2:**
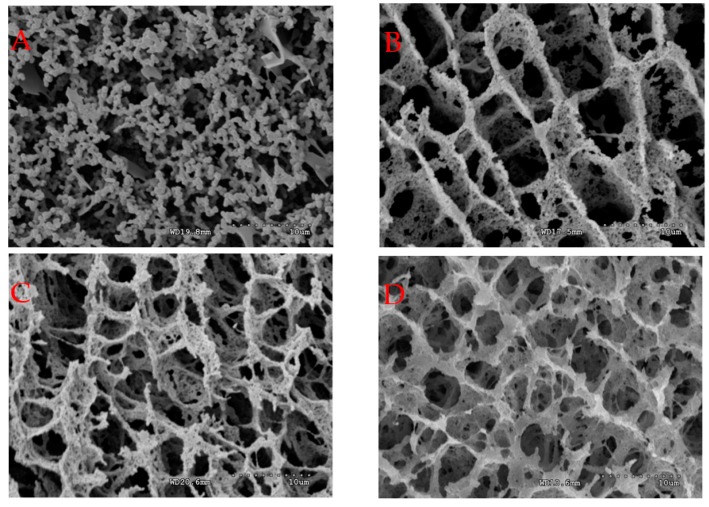

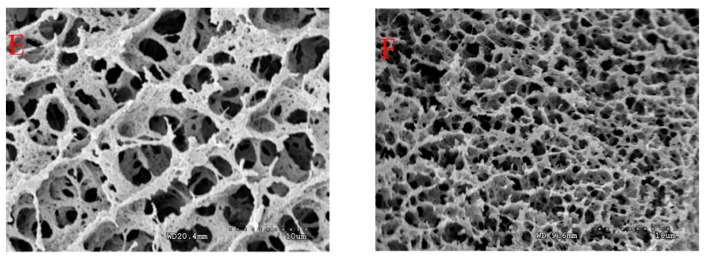
Cryo-scanning electron microscopy images of gels prepared from casein particle dispersions ((**A**) without mTGase and trisodium citrate, (**B**) With mTGase but without trisodium citrate, (**C**) TG-10 in route 2, (**D**) 10-TG in route 1, (**E**) TG-30 in route 2, and (**F**) 30-TG in route 2). Magnification = 3000×.

The microstructures of acid-induced casein gels prepared in route 1 and route 2 are shown in Figure 2. It can be clearly seen that gels prepared in route 1 had a more interconnected network than gels prepared in route 2 at the given concentration of trisodium citrate. In addition, the gels prepared in route 1 had smaller pores and more interconnected networks were present in the gels at 30 mmol/L trisodium citrate than at 0 mmol/L trisodium citrate. These trends align with the firmness and rheological results.

The cross-linking degrees of TG, TG-10, TG-20, and TG-30 were 19.9 ± 1.5%, 17.9 ± 0.5%, 21.2 ± 0.4%, and 21.7 ± 2.3%, respectively. This indicated that the trisodium citrate concentration in route 2 had little influence on the cross-linking degree of casein gels. The cross-linking degrees of TG, 10-TG, 20-TG, and 30-TG were 19.9 ± 1.5%, 29.8 ± 1.7%, 52.8 ± 1.6%, and 54.3 ± 1.9%, respectively. This indicated that, in route 1, the cross-linking degree of casein gels increased with trisodium citrate concentration (0 mmol/L to 20 mmol/L). As mentioned above, calcium-chelating agents can dissociate the native casein micelles into smaller particles [17]. This may have enhanced the contact between mTGase and caseins, which then increased the crosslink bonds between casein molecules before or during acidification.

### 2.6. Proposed Mechanism

**Figure 3:**
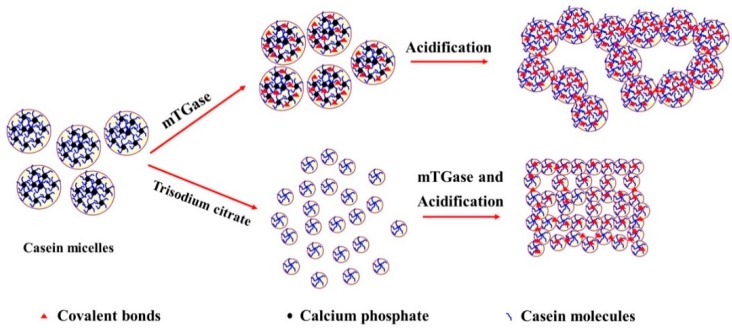
The schematic representation of the role of trisodium citrate in the formation of acid-induced, transglutaminase-treated casein gels.

The results presented above allowed us to explain the combined effect of trisodium citrate and mTGase on the gel properties of acid-induced micellar casein gels (Figure 3). In native casein micelles, the casein molecules are mainly cross-linked with colloidal calcium phosphate. In route 1, the citrate ion is a calcium chelating agent, which would disrupt the casein micelles by decreasing the [Ca^2+^] and [Mg^2+^] and colloidal calcium phosphate contents [27,34,35]. This favors the dissociation of native casein micelles into smaller casein particles [27]. Before acidification by GDL, the introduction of mTGase to the smaller casein particle dispersions resulted in partial cross-linkage of caseins within the particle matrix. In route 2, the introduction of mTGase to native casein micelles generated covalent protein networks. Trisodium citrate could bond to the calcium in the casein particles. However, the stability of the particle could not be disrupted due to the presence of covalent cross-linkage among casein molecules. This indicated that the flexibility of casein particles in route 2 was lower than in route 1. During acidification, the larger particles could not be adequately rearranged to form a more interconnected gel network. On acidification, due to the reduction of electrostatic repulsion, the casein particles began to aggregate. However, in route 1, due to their higher flexibility, the smaller casein particles were adequately rearranged to form more interconnected networks than the cross-linked native casein micelles. In this case, mTGase continued to crosslink two adjacent particles in the gel network even though the enzyme activity of mTGase gradually reduced with decreasing pH. Considering that the surface area of smaller casein particles was higher, the number of covalent bonds in gels in route 1 was larger than that in route 2, which led to denser and more stable gel networks. This may be the reason why the final G′ of gels prepared using route 1 was significantly higher than the G′ of gels prepared using route 2.

## 3. Materials and Methods

### 3.1. Materials

Micellar casein powder was purchased from Develing International (Bunschoten, The Netherlands). The contents of total solids, ash, fat, lactose, and protein in the micellar powder were 96.2%, 7.6%, 1.3%, 2.7%, and 85.6%, respectively. Calcium-insensitive mTGase was purchased from C&P Group GmbH (Rosshaupten, Germany) and the enzyme activity was 200 U/g. GDL, 2,4,6-trinitro-benzensulfonic acid (TNBS), and trisodium citrate were purchased from Sigma-Aldrich (St. Louis, MO, USA). All other chemical reagents used were of an analytical grade.

### 3.2. Preparation of Acid-Induced Micellar Casein Gels

#### 3.2.1. Preparation of the Micellar Casein or mTGase Dispersions

The micellar casein powder (2.5%, *w*/*v*) was hydrated in simulated milk ultrafiltrate (SMUF) at 55 °C for 40 min. During this process, the dispersion was stirred at 600 rpm. The SMUF was prepared based on a previously reported method [36]. The mTGase was hydrated in ultrapure water to a content of 4.0% (*w*/*v*) and stirred at 600 rpm for 1 h at 25 °C. The enzyme dispersion was further centrifuged (TDL-5C, Shanghai Anting Scientific Instrument Co. Ltd., Shanghai, China) at 3000× *g* at 25 °C for 15 min to remove the impurities at the bottom of the centrifuge tubes. The mTGase and casein micelle dispersions were stored at 4 °C prior to use.

#### 3.2.2. Pretreatment of Casein Micelle Dispersions

The casein micelle dispersions were pre-warmed to 42 °C, which was followed by the slow addition (with continuous stirring) of trisodium citrate powder into the dispersions to 0 mmol/L, 10 mmol/L, 20 mmol/L, and 30 mmol/L, respectively. For convenience, the citrate ions in the original SMUF were not included in the calculation of trisodium citrate concentration in this study. The dispersions containing different concentrations of trisodium citrate were magnetically stirred at 600 rpm for 5 min. The pH of the dispersions was adjusted to 6.6 with 0.5 mol/L of HCl. Afterward, a certain volume of the mTGase dispersions was added to the casein micelle dispersions. The final ratio of the mTGase enzyme activity to the mass of micellar caseins was 10 U/g. The samples were incubated in a water bath at 42 °C for 60 min, which was followed by acidification with GDL (as described in the following section).

For comparison, after mTGase treatment at 42 °C for 60 min, trisodium citrate-free casein micelle dispersions were added to trisodium citrate at concentrations of 10 mmol/L, 20 mmol/L, and 30 mmol/L, which was followed by acidification with GDL (as described in the following section). The trisodium citrate-free casein micelle dispersions in this paper refer to the dispersions without the addition of citrate before enzyme cross-linking of caseins even though SMUF contained approximately 10 mmol/L citrate ions.

For another comparison, micellar casein dispersions treated with 0 mmol/L trisodium citrate, 10 mmol/L trisodium citrate, 20 mmol/L trisodium citrate, and 30 mmol/L trisodium citrate (in the absence of mTGase) were also investigated.

**Figure 4:**
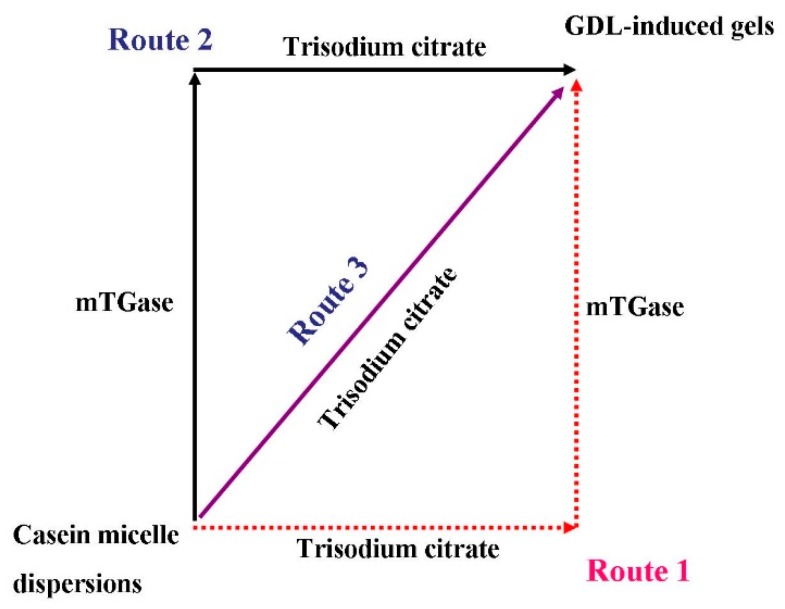
The schematic representation of the gel preparation route.

In summary, the preparation of casein micelle dispersions can be divided into three routes, which are shown schematically in Figure 4.

#### 3.2.3. Preparation of Acid-Induced Casein Gels

Following incubation, GDL was directly added to the dispersions under magnetic stirring (600 rpm) for 3 min at 25 °C. Afterward, the samples were left to stand in a water bath at 43 °C for 4 h at a pH of 4.3–4.6. The concentrations of GDL in the different dispersions prepared in Section 3.2.2 were 1.3% (*w*/*v*). The casein gels in the beaker were cooled by ice water and stored at 4 °C for further analysis.

#### 3.2.4. Characterization of Casein Micelle Dispersions or Gels

The sizes of the casein particles were determined using a dynamic light scattering (DLS) instrument (Nano ZS, Malvern, UK) at 20 °C. The treated samples were diluted 500 times with water [37]. Separation of calcium in the soluble and micellar phases was carried out by ultracentrifugation at 80,000× *g* for 3 h at 20 °C (CP 80MX, Hitachi, Tokyo, Japan) [34,38,39]. The calcium concentration in the soluble phase was analyzed by atomic absorption spectrometry (725-ES, Varian, Palo Alto, CA, USA).

The firmness of the casein gels was measured using a texture analyzer (TA-XT Plus, Stable Micro Systems, Godalming, Surrey, UK) in a single compression cycle test with a 5 kg load cell [40]. The set gels from cold storage (4 °C) were incubated at 25 °C for 60 min. The forces that pushed the cylindrical probe vertically into the gels (to a depth of 20 mm) at a speed of 60 mm/min were determined. Data were collected and analyzed to determine peak compression force.

The gel formation was determined using an MCR302 rheometer (Anton-Paar, Graz, Austria), based on the reported methods with some modifications [41]. An aliquot (15 mL) of the casein dispersions prepared in Section 3.2.2 (containing 1.3% GDL) was transferred to a concentric cylinder. A droplet of soybean oil was added onto the surface to prevent water evaporation. The gels were oscillated at a frequency of 0.1 Hz with an applied strain of 1% which was tested to be within the linear viscoelastic region. The test temperature was kept at 43 °C. Gelation time was defined as the point when the storage modulus (G′) of acid-induced gels ≥ 1 Pa [42].

The water holding capacity (WHC) of casein gels was determined based on a modified procedure [43]. A total of 8 mL of the pretreated casein micelle dispersions was poured into a 10 mL centrifuge tube and was acidified, which is described in Section 3.2.3. After acidification, the gels (Y) were centrifuged at 2800× *g* for 3 min at 25 °C. The mass of expelled water (W) was carefully collected and weighed. The WHC was calculated based on the formula below.

WHC = (Y − W)/Y × 100%(1)

The cross-linking degree of casein gels was measured based on a previously reported method with some modifications [44]. The final gels were mashed and washed with ultrapure water four times and then the samples were freeze-dried to a constant weight. One mL of 4 % (*w*/*v*) NaHCO3 solution and 1 mL of 0.1% (*w*/*v*) TNBS solution were added to the freeze-dried casein gels (5 mg). Following incubation at 40 °C for 3 h, 6 mol/L HCl (2 mL) was added to the samples and the temperature was raised to 60 °C. After reacting for 1.5 h, the samples were diluted with water (5 mL). The absorbance of the samples was determined at 345 nm using a U-2900 spectrometer (Hitachi, Ltd., Tokyo, Japan). The cross-linking degree (DE) was calculated based on the formula below.

DE% = (1 − (A_S_/m_s_)/(A_NS_/m_Ns_)) × 100% (2)

where A_S_ and A_NS_ represent the absorbance of samples and non-cross-linked casein micelles, respectively. m_s_ and m_Ns_ represent the mass of samples and non-cross-linked casein micelles, respectively. To remove the ions, the non-cross-linked casein micelles were acidified at a pH of 4.6 and freeze-dried to a constant weight.

The microstructures of the gels were observed by using S-3000N cryo-scanning electron microscopy (Hitachi Co., Tokyo, Japan), which was described in previous reports [45] with some modifications. Gels were deposited in the cryo-specimen holder (Quorum PP3000T, East Grinstead, UK) and fixed in slush nitrogen (<180 °C). After being fractured, the gels were sublimated at −85 °C for 35 min and sputter-coated with gold.

#### 3.2.5. Statistical Analysis

The experiments were carried out in triplicate. The means and standard deviations were calculated from these measurements. Data were tested using a two-way analysis of variance in order to determine the differences in particle size, firmness, and water-holding capacity induced by routes and trisodium citrate concentrations. SPSS 18 software for Windows (IBM, Ehningen, Germany) was used as the statistical analysis software. Duncan’s test was applied to determine the statistical differences between the means.

## 4. Conclusions

In this study, the effect of trisodium citrate on the textural properties and microstructure of acid-induced, transglutaminase-treated micellar casein gels was investigated. It was concluded that the casein micelles that were first treated by trisodium citrate (10–30 mmol/L) and then cross-linked with mTGase (route 1) resulted in more stable acid gels than those directly cross-linked with mTGase (route 2) or without mTGase (route 3). The trisodium citrate (10–30 mmol/L) could dissociate the native casein micelles into smaller casein particles. In the presence of mTGase, the smaller particles formed acid-induced gels with greater firmness, higher water-holding capacity, more interconnected networks, and smaller pores than native casein micelles.

At the present time, mTGase is listed as a food ingredient in several commercial acid-induced milk gel products sold in many countries. It is used to improve the textural properties of acid-induced gel products. However, textural defects such as low firmness (fragile structure) and syneresis cannot be completely avoided. The method proposed in route 1 provides a novel strategy for improving the textural properties of acid-induced milk gel products.

## Figures and Tables

**Table 1:** The effects of trisodium citrate on the average diameter (nm) of casein particles ^1^.

Treatment	Trisodium Citrate Concentration (mM)			
	0	10	20	30
Route 1	144.0 ± 6.0 ^a,A^	88.3 ± 2.9 ^b,A^	24.7 ± 1.7 ^c,A^	18.8 ± 1.8 ^c,A^
Route 2	144.0 ± 6.0 ^a,A^	129.7 ± 0.8 ^a,B^	130.3 ± 1.2 ^a,B^	137.4 ± 2.2 ^a,B^
Route 3 (Without mTGase)	142.2 ± 2.2 ^a,A^	79.4 ± 9.5 ^b,A^	20.3 ± 2.9 ^c,A^	13.6 ± 1.6 ^c,A^

^1^ Superscripts with different letters (a–c) and (A, B) for the same row and column, respectively, indicate significant differences (*p* ≤ 0.05).

**Table 2:** The effects of trisodium citrate on the firmness (mN) of casein gels ^1^.

Treatment	Trisodium Citrate Concentration (mM)			
	0	10	20	30
Route 1	86.7 ± 6.1 ^a,A^	114.8 ± 1.3 ^b,B^	175.2 ± 1.3 ^c,B^	179.4 ± 8.9 ^c,C^
Route 2	86.7 ± 6.1 ^a,A^	68.9 ± 1.6 ^b,A^	65.0 ± 1.4 ^b,A^	61.8 ± 1.9 ^b,A^
Route 3 (Without mTGase)	67.8 ± 3.1 ^a,A^	65.7 ± 5.7 ^a,A^	71.5 ± 2.5 ^a,A^	69.7 ± 1.2 ^a,B^

^1^ Superscripts with different letters (a–c) and (A–C) for the same row and column, respectively, indicate significant differences (*p* ≤ 0.05).

**Table 3:** The effects of trisodium citrate on the water holding capacity (%) of casein gels ^1^.

Treatment	Trisodium Citrate Concentration (mmol L^−1^)			
	0	10	20	30
Route 1	65.4 ± 5.2 ^a,A^	95.4 ± 0.8 ^b,B^	98.7 ± 0.1 ^c,B^	97.7 ± 0.2 ^c,B^
Route 2	65.4 ± 5.2 ^a,A^	59.4 ± 4.1 ^a,A^	66.0 ± 2.3 ^a,A^	64.8 ± 2.2 ^a,A^
Route 3 (Without mTGase)	58.6 ± 1.3 ^a,A^	63.5 ± 0.7 ^b,A^	61.4 ± 1.6 ^a,A^	60.1 ± 1.7 ^a,A^

^1^ Superscripts with different letters (a–c) and (A, B) for the same row and column, respectively, indicate significant differences (*p* ≤ 0.05).
